# The multi-domain responder index: a novel analysis tool to capture a broader assessment of clinical benefit in heterogeneous complex rare diseases

**DOI:** 10.1186/s13023-021-01805-5

**Published:** 2021-04-19

**Authors:** P. K. Tandon, Emil D. Kakkis

**Affiliations:** 1 Ultragenyx Pharmaceutical Inc., Novato, CA USA; 2 Ultragenyx Gene Therapy, 840 Memorial Drive, Cambridge, MA 02139 USA

**Keywords:** Multi-domain responder index, MDRI, Mucopolysaccharidosis, MPS, Laronidase, Vestronidase alfa

## Abstract

In traditional clinical trial design, efficacy is typically assessed using a single primary endpoint in a randomized controlled trial to detect an expected treatment effect of a therapy in a narrowly selected patient population. This accepted paradigm is based on clinical evaluations that may not actually capture the breadth of the impact of a disease, which is especially true in the setting of complex, multisystem, rare diseases with small, extremely heterogeneous patient populations. The multi-domain responder index (MDRI) is a novel approach that accommodates complex and heterogeneous disease manifestations and evaluates a broad array of clinical disease without impairing the power or rigor of a study to fully understand a treatment. The MDRI sums the scores corresponding to clinically significant thresholds of change for each component domain in each individual patient, capturing the mean clinically meaningful change across multiple domains within individuals. This novel approach combines and then sums the results of independent domain endpoint responder analyses into one responder score to provide a broad basis for the assessment of efficacy. The impact of a treatment across multiple, physiologically independent domains, can be assessed clinically, reducing the adverse impact of heterogeneity on trial outcomes and allowing eligibility criteria to enroll a wider range of patients, ultimately resulting in efficacy and safety assessments of a therapy across a broad group of heterogeneous patients in rare disease programs.

*Trial registration* The following studies are referenced within this manuscript (CLINICALTRIALS.GOV registration numbers): NCT00912925; NCT00146770; NCT00067470; NCT00104234; NCT00069641; NCT02230566; NCT02377921; NCT02432144.

## Background

In traditional clinical trial design, efficacy is most often based on rejection of the null hypothesis using a single primary endpoint in a randomized controlled trial. These studies are specifically designed to enhance the detection of the expected therapy treatment effect by assessing one efficacy dimension in a primary evaluation of a narrowly selected patient population. This study design and analysis paradigm misses the opportunity to study a broader patient group because of the need to select specific patients for the chosen primary endpoint. The clinical validity of this approach has been assumed since the beginning of efficacy requirements in drug development as a part of the regulatory approval process that began in the United States with the Kefauver-Harris Drug Amendments in 1962 [1].

This accepted paradigm is not based on clinical evaluations that may actually capture the breadth of the impact of a disease. Patients and doctors rarely, if ever, cite a single measure of disease or function as an indicator of drug efficacy, yet the system for investigational agent study design and regulatory approval has rested solidly on this approach.

Depending on the study design, different observers may have different views of the same disease, like seven different blind doctors studying an elephant. There is a need to reshape our clinical efficacy determination approach to better capture the breadth of study drug effectiveness across a wider spectrum of patients and provide insight into multiple clinical domains. We are proposing a novel approach to achieve a multi-domain clinical response measurement and analysis, the multi-domain responder index (MDRI). The MDRI enables a comprehensive evaluation of all-comer rare disease studies to provide a broader and deeper insight into the impact of a therapy with substantially greater power and high rigor, while also allowing a more complete analysis of safety across a range of patients closer to real world exposure.

## Main text

### Key challenge in the analysis of randomized trials in rare diseases: extreme heterogeneity

In diseases with large patient populations, study designs that provide adequate power to detect a treatment effect difference in a single primary endpoint as well as secondary endpoints are possible through the inclusion of hundreds of carefully selected study subjects among many thousands available. In rare diseases with small patient populations, extreme heterogeneity makes it difficult to find enough patients and to power more than a primary endpoint, except in the most homogeneous diseases with closely related endpoints [2, 3]. For rare diseases, it is common and often essential to select a single primary endpoint in a carefully selected population that has a baseline level of disease with an assessable primary endpoint. Study subjects should be of an age where therapeutic efficacy to reverse the course of the disease is still possible. Eligibility criteria should also include characteristics that consider the ability of the subject to perform the primary endpoint assessments. Powering secondary endpoints that are independent, and not derivative, of the primary endpoint is not typically possible in studies with small sample sizes. In rare diseases, selection of a study population with both primary and secondary endpoint characteristics optimum for evaluation oftentimes results in a net eligible population that is so small, it is nearly impossible to enroll. Because it is difficult to incorporate secondary endpoint characteristics into patient selection criteria, there is a tendency toward a one-dimensional assessment of disease that is often not fully correlated with patient self-perception of health improvement. When power to detect differences in secondary endpoints is reduced due to rare disease heterogeneity, there may be a misperception that a treatment effect is not happening, when, in fact, the problem being assessed is not present at baseline in many patients. This type of situation presented itself in the Phase 3 study of laronidase enzyme replacement therapy for mucopolysaccharidosis type I (MPS I) in which one co-primary endpoint was achieved and the second co-primary endpoint was missed due to too much baseline variation in the study population, and three secondary endpoints failed to indicate a treatment effect difference in the intent-to-treat population due to 50% or fewer patients expressing the abnormality at baseline [2].

#### The impact of heterogeneity and patient selection in the Phase 3 Laronidase study

The challenges associated with defining a patient population and endpoint evaluation are best illustrated using data from the Phase 3 laronidase study in patients with MPS I, a devastating, multisystem disorder with highly variable symptoms, including airway obstruction, pulmonary disease, cardiac disease, hepatosplenomegaly, small joint restriction, shoulder restriction, skeletal disease, brain abnormalities, corneal clouding, and decreased visual acuity. Laronidase is an enzyme replacement therapy that was shown in a dog model to deliver enzyme to a broad array of tissues and was expected to impact multiple diverse domains of the disease [4].

Given the regulatory agreed upon co-primary endpoints of forced vital capacity (FVC) and the six minute walk test (6MWT), a clinical survey study was conducted that showed that only 25% of patients would be substantially impaired in both measures at the same time. This finding suggested that enrollment and conduct of the study would be feasible if eligible patients were selected based on only one of the assessments having abnormal measures at baseline. With that limitation, FVC impairment of less than 80% of normal was selected as an eligibility criterion, and no impairment in 6MWT was specified for eligibility to allow enrollment of sufficient patients to enroll the study. Secondary endpoints included the Sleep Apnea–Hypopnea Index (AHI) and Shoulder Flexion range of motion (ROM), with no eligibility restrictions for these parameters, and while impairment is common, it was not known exactly how many subjects would show the abnormality at baseline but that did show benefit in the Phase 1/2 laronidase study. Visual acuity was a tertiary endpoint for which improvement in affected patients had also been observed in the Phase 1/2 laronidase study.

#### High level summary of the Phase 3 Laronidase study: conventional analysis of the co-primary endpoints

The study enrolled 45 subjects, aged 6–43 years, randomized 1:1 to drug versus placebo and treated for 6 months of weekly infusions. Less than 50% of subjects were impaired sufficiently in the 6MWT at baseline to be able to reliably reveal improvement. Other than the nonspecific ability to walk at least 50 m, there was no eligibility criterion specific to the rigors of the 6MWT. The baseline data from this study showed substantial patient heterogeneity in 6MWT results with the ranges at baseline of 60–571 m for the placebo group and 14–591 m for laronidase group. The median baseline 6MWT distance was close to 350 m for both the placebo and laronidase groups, near the lower limit of normal for healthy adults, meaning only about half the subjects were more significantly impaired. These baseline ranges are approximately tenfold the magnitude of a clinical meaningful change of 53 m used in evaluating responders in this study, making it difficult to detect a clinically meaningful change.

As expected, after the study was unblinded, FVC showed a statistically significant improvement of 5.6 percentage points in percent of predicted normal FVC (median, 3.0; *p* = 0.009) with laronidase treatment; however, given the mean FVC was around 50% of normal at baseline, the relative change was > 11%, a clinically meaningful difference. In the double-blind portion of the trial, patients receiving laronidase showed a mean increase in the 6MWT distance whereas placebo-treated patients showed a decrease, for a difference between groups of 38.1 m in 6MWT distance (median, 38.5). Using the intent-to-treat (ITT) analysis set and the primary specified analysis (Wilcoxon rank sum test), this difference approached, but did not reach, statistical significance (*p* = 0.066). If an analysis of covariance (ANCOVA) was used to control for baseline walk distance and age, the result was *p* < 0.039 [2].

In the open-label extension portion of this trial, subjects who continued on laronidase showed an additional 20 m mean increase in 6MWT distance. The mean change from baseline of nearly 40 m was statistically significant (*p* = 0.005). The placebo crossover subjects confirmed the treatment effect, as their increase in 6MWT distance was of a similar magnitude to the laronidase-treated subjects in the double-blind phase, and the difference in 6MWT distance from crossover at week 26 to the end of study was also statistically significant (*p* = 0.023). These data, other data from the study, and data from a long-term extension study confirmed the impact on disease as measured by the 6MWT test observed in the double-blind 6 month period of the study [2, 5]. Laronidase does improve the 6MWT, but with the heterogeneity at baseline, this was missed by the primary analysis method.

#### High level summary of the Phase 3 Laronidase study: analysis of secondary endpoints

If we assume the alternative analysis for the 6MWT, then results from the 6MWT and FVC were positive for laronidase-treated subjects; however, three other endpoint domains, Shoulder Flexion ROM, Sleep AHI, and Visual Acuity, were negative, but only a subset of ~ 50%, ~ 50%, and ~ 18% of subjects were abnormal in these three measures at baseline, respectively. The negative outcomes were likely due to the heterogeneity of baseline disease. Not surprisingly, the statistical analysis plan used the requisite ITT approach for these endpoints, leading to the dilution of treatment effect by those unaffected by the problem and the loss of power to detect a statistical difference in affected subjects.

Shoulder Flexion ROM had shown a strong positive result in an earlier Phase 1/2 study in 10 patients, all of whom were substantially impaired with restricted shoulders (usually less than 90° flexion). In that study, Shoulder Flexion ROM showed a statistically significant improvement of a mean 26–28 degrees. Given the earlier result from the Phase 1/2 study, it was unexpected that this domain would be affected in only 50% of subjects and that the observed improvement would not achieve a statistically significant difference in the ITT population directly due to this heterogeneity. In a subset analysis of patients with ROM < 90.5° (the study median), laronidase-treated subjects had a mean improvement of 9.6° versus placebo-treated subjects with a mean decline of 4.8° [2].

Similar to the results observed in the Shoulder Flexion domain, approximately 50% of patients at baseline had sleep apnea, defined as AHI > 10. Again, this endpoint had shown a strongly positive result in two patients with sleep apnea in a phase 1/2 study and missed achieving a statistically significant difference in the phase 3 ITT population, though a mean overall improvement in Sleep AHI was observed. In a subset analysis of patients with minimum or greater severity considered clinically significant (AHI ≥ 10 for subjects ≤ 15 years and AHI ≥ 15 for subjects > 15 years at baseline), laronidase-treated subjects had a mean decrease of 6.0 events per hour of sleep versus placebo-treated subjects with a mean increase if 0.3 events per hour of sleep, a statistically significant difference (*p* = 0.014) [2].

For the Visual Acuity domain, only 8 of 45 patients (18%) were visually impaired at baseline, with acuity of ≥ 20/200. This domain missed achieving statistical significance due to the small number of patients with visual impairment at baseline, though in five laronidase-treated patients, visual acuity improved substantially by 4 lines from 20/200 to 20/50 or better. Notably, in the three placebo-treated subjects with visual acuity impairment at baseline of ≥ 20/200, acuity did not change during the study.

These data from the laronidase Phase 3 study highlight one of the major limitations of analyzing endpoints by mean change from baseline in the ITT population that patient heterogeneity dilutes the impact of any effect observed. A second limitation is that the results are presented by endpoint, not by patient, making it difficult to know for a multisystem disease, like MPS I, how an individual patient has responded to treatment in an integrated fashion.

## Summary of key learnings from the Phase 3 Laronidase study

The laronidase Phase 3 trial is one example of this adherence to historical study design and endpoint analysis, a pattern repeated in many other rare disease programs. Due to limited numbers of rare and ultra-rare disease patients, it is always difficult to make studies large enough to power all endpoints, leading to the focus on the primary endpoint and the appropriate population for that endpoint. The substantial and necessary enrollment criteria of rare disease pivotal trials for disease severity, age, ability to conduct the study assessments, and other factors to focus on the primary endpoint unintentionally impairs our capacity to understand the true breadth of the therapeutic efficacy of an investigational agent. Overly restrictive eligibility criteria focused on selecting patients for all endpoints usually leads to slow enrollment or no enrollment, whereas eligibility criteria powered to detect a difference for the primary endpoint of the study may lead to the dilution of effect on secondary endpoints previously described. Often, a wide age range must be included to find enough patients, making interpretation of data quite complex, as exemplified from the laronidase trial above, where 6MWT results had to be interpreted for subjects ranging in age from 6 to 43 years [2].

### The case for why multiple domain evaluation is a more suitable clinical approach in rare disease drug development

While it is clear that smaller study sample sizes and narrowly selected patient populations compromise the ability to conduct efficient rare disease studies, the clinical validity and value of single primary endpoints as a research approach could be questioned. More precisely, we need to assess if our single primary endpoint designs truly capture the breadth of efficacy as measured by how a patient feels and/or functions after treatment. Most people would naturally believe that any individual patient is feeling many different domains at once and integrate that feeling to provide a sense of their health. If we use a single endpoint or three endpoints, the question is how predictive of a patient’s health assessment are those data and therefore which approach is a better, more complete assessment of efficacy? The accuracy of this correlation will tell us how valid a single endpoint assessment captures clinical benefit and if multiple independent domains more accurately predict and capture clinical benefit.

To assess the relative predictive value of individual endpoints versus multiple domains for an integrated assessment of patient self-health, the EveryLife Foundation in its first workshop in 2010, prepared a correlation analysis with data from three randomized placebo-controlled Phase 3 studies of enzyme replacement therapy in MPS diseases [2, 3, 6]. The analysis of trial data from studies of laronidase, idursulfase, and galsulfase for MPS I, II, and VI, respectively was overseen by statistician James Signorovitch and The Analysis Group in Boston. Correlations were conducted singly and as a group between the Child Health Assessment Questionnaire (CHAQ) or the Health Assessment Questionnaire (HAQ) administered to adults (an integrated measure of perceived health by the patient), and the 6MWT, FVC, and shoulder flexion ROM singly and in combination. Patient assessments were pooled from the treatment and placebo arms of these trials (N = 76). The Pearson correlation was calculated comparing the change in 6MWT, FVC, and Shoulder Flexion ROM with the CHAQ/HAQ scores and determined to be − 0.24, − 0.19, and − 0.23, respectively, with *p*-values in the 0.03– 0.09 range (Table 1). These results were consistent with a modest correlation between any single endpoint and the CHAQ/HAQ scores and was consistent with the positive results. To combine the three endpoints, a summation of rank scores for each point was analyzed using the O’Brien t-test. The O’Brien rank sum score composite of three clinical endpoints was more strongly correlated with patient-reported health, accounting for ~ 50% of treatment-related change with perceived health than each of the analyzed endpoints individually, indicating the clinical utility, and more importantly, the powerful validity, of capturing more domains in the total assessment than could be achieved with a single primary endpoint (Table 1).

**Table 1:** Pearson correlation with CHAQ/HAQ for individual and O’Brien rank score of three clinical endpoints

Assessment	Pearson correlation with CHAQ/HAQ^a^, N = 76	*p* value
6MWT	− 0.24	0.032
FVC	− 0.19	0.091
Shoulder ROM	− 0.23	0.051
O’Brien rank score (6MWT, FVC, Shoulder ROM)	− 0.50	< 0.001

*6MWT* six minute walk test, *FVC* forced vital capacity, *ROM* range of motion

^a^Patient assessments pooled from treatment and placebo arms. Higher CHAQ/HAQ scores indicate greater impairment

These data suggest that patient perception of self-health is nearly equally impacted by all three endpoints, leading to a near summation of the correlation rate in the three individual endpoint analyses. As most clinicians and patients know a priori, these data support the concept that if a greater number of different problems get better, you feel much better than you do if only one problem is better. Implicit here is that each patient might contribute a different outcome for each endpoint to the analysis, but the combination gives a better and more valid clinical answer than an individual patient alone.

### Composite endpoints or disease scores do not usually solve the problem of sensitivity in the complex, multi-domain setting in the face of heterogeneity

Composite endpoints for efficacy assessments are limited in sensitivity in heterogeneous patient populations and are best applied when the composite is based on a single pathophysiologic concept or highly correlated set of domains that have been clinically validated [7, 8].

A composite analysis, as in the O’Brien analysis of rank sum summary, is one approach to gaining power from multiple domains, but composites calculated in this way or other ways can have fundamental challenges in that the magnitude of effects being added are not necessarily known and might win on the ranking but lack clinical meaningfulness. Many rare disease groups also try to resolve the challenge of combining domains by creating composite disease scores that are comprehensive additions of many small subscores. The main factor driving the use of composite endpoints or scoring systems in multisystem and rare disease is to either increase the number of expected events or expand the sensitivity to detect changes in more endpoints, thereby increasing the power to detect efficacy and reducing the required total sample size. However, the use of composite scores across many domains often leads to the dilution of benefit or decline in one domain that may be attributable to variation in other domains due to individual patient heterogeneity. So if one domain has a very substantial benefit and gains in the score, random variation in two or three other subscores in that individual can readily dilute the effect and lose the thread of efficacy in the noise of too many disparate scores.

Composite endpoints, in general, are more likely valid when they are based on a common single pathophysiological concept, such as coronary artery disease and the composite of myocardial infarction, stent, coronary artery bypass grafting, and cardiac death [9]. The North Star Ambulatory Assessment (NSAA) is an example of an effective composite endpoint that combined a highly correlated, single functional domain set of muscle-dependent, physical performance scores [10]. Similarly, the composite muscle strength of four muscles in the upper extremity measured using handheld dynamometry has been shown to be a valid estimate of upper extremity physical function predictive of patient ability to do common tasks of daily living, as developed for the disease GNE myopathy [11]. Composites of medical events, such as emergency room visits or hospitalizations, can also be effective, even when the source of underlying causes of the medical events might be variable or due to multi-domain complications, such as in the case of major clinical events in patients with long-chain fatty acid oxidation disorders, where complications in the liver (hypoglycemia), heart (cardiomyopathy), or skeletal muscle (rhabdomyolysis) all act as independent physical causes that can lead to the common result of hospitalizations [12]. This has also been successfully used recently in acute intermittent porphyria that has CNS, abdominal pain, and variable forms of crises as reasons for hospitalizations [13].

In diseases with extreme heterogeneity and multiple domains with different pathophysiologic concepts that lack a common final pathway outcome, the composite multi-domain endpoint approach that is scored in total, per patient, will cause dilution of efficacy and lead to insensitive, less rigorous results that can be very complex to understand, given the mixture of outcomes and pathophysiology contributing to the result [7]. Developing and validating a novel composite endpoint also requires more studies, time, and subjects than is possible with most rare diseases. Even when possible to do, clarity on interpretation of scoring, validation of within-patient summations, and weighting of results to understand a clinical profile of a therapy is nearly impossible in most rare disease populations.

A method that (1) does not require prior validation of the composite, (2) allows individual domain analysis and scoring on an individual patient basis, and (3) combines clinically meaningful results afterward, could reduce the dilution of efficacy and any impact heterogeneity may have on the interpretation of results and succeed with fewer assumptions about who is enrolled. If scoring for each individual domain is based on accepted or known minimally important clinical differences, we might also avoid the concept that we are adding up inconsequential changes to make a consequential result, a criticism that might be levied against the O’Brien rank sum method despite the value of this methodology shown in the analysis above. With the use of the new analytical tool called the multi-domain responder index (MDRI), the impact of a treatment across multiple physiologically independent domains can be assessed clinically, and the adverse impact of heterogeneity can be reduced, thereby allowing broader eligibility criteria to enroll a wider range of patients with fewer assumptions about who will enroll, ultimately resulting in an increased breadth of efficacy and safety assessments in rare disease programs.

### The MDRI as a novel and powerful approach for rare disease study analysis

The MDRI was initially conceived as a novel way to address the clinical heterogeneity often observed in patients in multi-system diseases and their heterogenous responses to treatment across different domains. This idea came from the O’Brien global test statistics which was further expanded by Pocock et al. and applied by Tandon [14–16]. This non-parametric test combines the rank sums of several domains within the same patient and then compares the differences before and after treatment between groups. Instead of rank sums, the MDRI sums the scores corresponding to clinically significant thresholds of change for each component domain in each individual patient, using the minimally important difference established as the measuring stick for the threshold. This way MDRI captures the mean clinically meaningful change across multiple domains within individual patients. In summary, this is a novel approach that combines then sums the results of independent domain endpoint responder analyses into one responder score to provide a broader basis for the efficacy assessment.

Each of these individual domain endpoints within an MDRI should represent relatively distinct clinical domains with potentially unique pathophysiologic bases and are scored independently, first using clinically-based criteria for a minimally important difference (MID) to calculate a responder score + 1, 0, or − 1 MID change. These independent domain results are then combined as individual patients and as a group to determine the net domain improvements or declines observed in each treatment group.

The endpoint types and the scoring of these endpoints individually as responder analyses is a very traditional practice, but the key difference in this case is that independently, domain results are scored individually by patient first, before combining results. This allows a subset of patients with a less common problem that substantially improves to contribute to the overall efficacy assessment by scoring tabulated clinically meaningful responses to the total net score. Scoring each domain first in each patient also prevents the dilution of effects from one patient affected by a problem by the others that are not. The MDRI analysis method evaluates large individual patient changes that exceed the MID and count to a score, while noise resulting from smaller changes is filtered out. Patients without a specific problem do not impact the analysis: “0” results drop out in the analyses, so heterogeneity does not impact efficacy results, and patients with irreversible disease or domains that cannot change or be assessed do not change the interpretation of results. This approach also preserves the ITT principle as all patients are included in the analysis.

Importantly, individuals that decline substantially in certain domains are not ignored but count against improvements in other domains. Unlike composite endpoints, where the combination of arbitrary scores have more substantial issues of weighting and balance, as well as the dilution effect, the use of the MID measuring scale on each endpoint allows a measure of confidence that important changes are being quantified for each endpoint before they are added. Only changes that meet or exceed the MID threshold are added, and insignificant changes do not impact the overall assessment. It also assures that for a given endpoint, scoring only happens with a clinically meaningful change. The MDRI avoids some of the complexity associated with constructing and validating composite endpoints, which is often not possible in rare diseases.

**Fig. 1:**
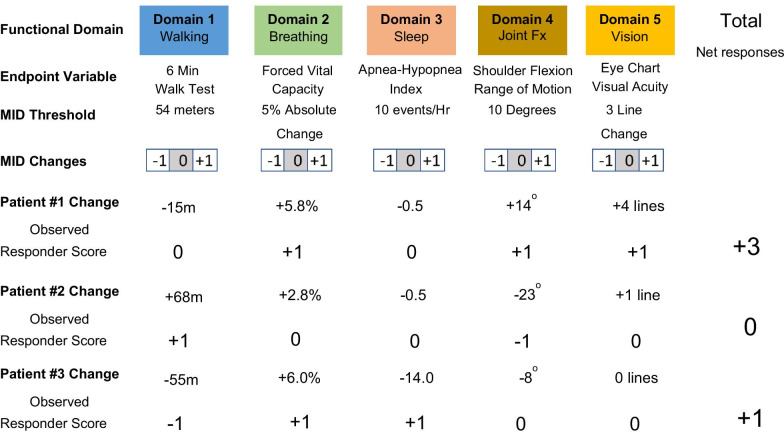
MDRI example construction and calculation

Application of the basic MDRI analysis is relatively simple and an example will help clarify the method (Fig. 1). First, a set of clinically important domains should be defined using patient surveys, interviews, and clinical surveys to establish their importance. Next, endpoints are defined that represent those domains, but with the requirement that the different domain evaluations optimally be relatively independent of each other, and not duplicative. Among the set of endpoints in a study, the MDRI analytical strategy would choose a priori, four to six unique, relatively independent domain endpoints of clinical relevance that cover the disease in a range of patients. In the example in Fig. 1, we chose domains and endpoints that were part of the laronidase clinical study of MPS I. It would not be expected or required that all patients would be able to score in all domains, such as a case where a patient cannot do the test (*eg*, a walk test in a person who cannot walk) or where the problem assessed is not present at baseline (*eg*, a person who can already walk a normal distance and therefore cannot really walk much farther during treatment).

By scoring each patient using a clinically relevant magnitude cutoff, such as the MID, each patient is scored using assessable endpoints, with not assessable or unchanged domains receiving a score of 0, which does not impact the overall assessment. After scoring individual patient responses in each MDRI domain as shown, we can see how individual patients fared in each domain and how any declines in domains deducted from any positive benefit. We can then add the presence of clinically meaningful scores to obtain an effective net change assessment of the totality of the clinical efficacy data. When analyzing data, one can summarize net domain changes and compare results from a control group using a Student’s t-test. Alternatively, an evaluation of the ratio of improvement versus decline domains can provide insight into the net shift of the treated population toward overall improvement or toward decline. While more power might be obtained using a linear parametric analysis for each endpoint, the loss of power using the responder-based scoring is more than compensated for by the power of the additional orthogonal endpoint evaluations. Responder scoring also improves the interpretability, intrinsic clinical meaningfulness, and clarity of the combined results than using continuous variable results. The MID filter also reduces small methodologic and biologic variations from the analysis and can help reduce biologic or procedural/test noise within the context of rare disease studies in small patient populations.

The MDRI is intended to capture the aggregate benefit or decline across multiple domains of clinical function to assess clinically important changes. Equal weighting for each domain is given, and none of the domains are judged to be more clinically important or more likely to show a benefit or decline than others. It is certainly possible to weigh each domain differently, but adding weights introduces substantial additional complexity. This also creates difficulties in interpreting what a fractional change in a domain would mean when the weighting applied is not an integer. The assumption that none of the domains are more clinically important or likely to show a benefit or decline than others is an approximation and as imperfect as it may be, the domains proposed for the analysis can be negotiated a priori with regulators to establish a set of domain endpoints that can be measured that are reasonably independent and potentially of similar though not identical value, even if subjective views make the exact mathematical equality of an endpoint value change essentially impossible to calculate. It is also unnecessary that values be exactly the same, as for any patient the relative meaning for any endpoint, and its change, can be different for the purposes of establishing substantial efficacy, an imperfect summation itself.

### Features of the MDRI method

#### Enrollment criteria can be broadened to allow a wider spectrum of subjects to enroll

Since patient heterogeneity does not alter the analysis using the MDRI because the MID lens filters out any lack of change in those without disease or irreversible disease or when assessments cannot be done, enrollment of patients with a wide range of baseline dysfunction and disease severity into a clinical trial is allowed, leading to the better characterization of the impact of a therapy in a broader patient population. Since each patient can score differently in distinct subsets of the domains, some patients may score in some domains but not be assessable in others, and this variability of assessment does not impact interpretation of the results. Importantly, all patients support the assessment of safety. All domains are scored, with those not assessable or unchanged scored as 0, which does not count for or against the patient outcome and overall assessment.

The MDRI assesses what is assessable, and there are no exclusions or penalties for the inability to perform an assessment. Restrictive eligibility criteria to filter out patients with high disease burden are unnecessary, allowing the MDRI to give a broader view of efficacy across a patient population. If very few assessments can be done in a population or in a given patient, power to detect differences is, of course, lost, but if variation occurs that harms the power of one or more endpoints, this variation may not impact the power of other assessments to gauge change. If the enrolled patient population unintentionally turns out different from an earlier hypothesis generating study, all is not lost because of this accidental variation, which is a common problem in rare disease studies, including the laronidase example on shoulder ROM.

#### The breadth of endpoints can be constructed to assure all patients have some assessments

While the presence of patients that cannot complete certain tests or do not have baseline disease have no effect on the outcomes assessed in a study, domains and endpoints can be chosen to assure that more severe patients have at least some assessable domains. For example, some patients may not be able to walk due to pre-existing hip degeneration for a disease, but they can use their hands and their shoulders, both of which are needed for activities of daily living. By assessing fine motor skills, shoulder function, and walking, both non-walkers and walkers can be assessed for clinically important physical function changes.

#### Intermediate or clinician or patient-reported endpoints can be used

Clinical physiologic measures, like pulmonary function testing, can be ideal endpoints because they are an objective test with established MIDs that directly measure function. Clinician or patient-reported outcome measures, like fatigue scores or global impression scales, can be incorporated into an MDRI score, provided that the properties of the test and the proposed MIDs have been established. In rare diseases, true validation of MIDs is essentially impossible since there are not enough patients to conduct the requisite testing; therefore, the MID must be based on other disease states. Work must then be done to qualify the magnitude and meaning of the MID as a valid domain for the assessment of the rare disease compared with the source data and disease that supported the MID.

### The value and application of the MDRI method in two MPS clinical studies

#### Use of an MDRI post-hoc analysis of the Phase 3 Laronidase study

To show how the MDRI might be evaluated within a clinical trial, we will illustrate the use of MDRI method in two studies in MPS diseases, laronidase for MPS I and vestronidase alfa for MPS VII. MPS diseases are a group of rare inherited lysosomal storage disorders that are debilitating, life-threatening, heterogeneous, and caused by a deficiency of one of the enzymes in stepwise degradation of complex carbohydrates known as glycosaminoglycans (GAGs). Every tissue and organ in the body is impacted by lysosomal storage and consequential tissue damage and inflammation and therefore serious outcomes in every domain are possible.

For laronidase, the MDRI analysis was first utilized in a post-hoc analysis of the Phase 3 study in a presentation to the Endocrinology and Metabolism Advisory Committee meeting to review the laronidase Biologics License Application on 15 January 2003. Using our knowledge of the disease from a clinical survey, patient interviews, and Phase 1/2 clinical data, a set of domains were established, and using the MID information, the data were analyzed in the heat map format.

As previously described, when examined individually, many of the secondary and other endpoints in this trial did not reach statistical significance primarily due to patient heterogeneity at baseline. Table 2 includes a column describing the prevalence of each abnormality by endpoint at baseline in the Phase 3 enrolled patients. For FVC, a pulmonary function test, 100% of subjects had less than 80% of normal predicted FVC at baseline since this was an entry criterion for the study. For the 6MWT, Shoulder Flexion ROM, AHI, and visual acuity, 50% or fewer patients were abnormal in each evaluation at baseline. In this study, the requirement for the FVC test at baseline led to the shift toward older, more capable subjects that could successfully do the pulmonary function test, and younger patients with more severe joint restriction ended up qualifying less often, so that shoulder range of motion that was nearly universally abnormal in Phase 1/2 with younger more severe patients (< 90° at baseline), was now present in only half of the Phase 3 enrolled subjects. This is a typical example of how the attempt to control one endpoint leads to inadvertent, unexpected, and adverse impacts on other endpoints. This is a common theme in rare disease studies. Trying to control study entry with eligibility criteria that address more than one endpoint would have resulted in a study that would be impossible to enroll.

**Table 2:** MDRI domains, MID thresholds, and study population from the Phase 3 Study of Laronidase

Domain	MID threshold	Study population with baseline deficiency in domain
6MWT	± 54 m	Approximately 50% < 350 m
FVC	± 11%	FVC < 80% was entry criterion; 100% qualify
Shoulder ROM	± 20°	Approximately 50% had ROM < 90°
AHI	± 10 events per hour	Approximately 50% AHI > 10
Visual acuity	± 2 lines	8 Patients (approximately 20%) > 20/200

*MID* minimally important difference, *6MWT* six minute walk test. *FVC* forced vital capacity, *ROM* range of motion, *AHI* apnea hypopnea index

An MDRI composite endpoint analysis was performed using five domains: the two co-primary endpoints, two secondary endpoints, and a tertiary endpoint. These domains aligned with the results of the Phase 1/2 study in which these domains were found to be both important and treatable. The MID for each domain was defined using the literature available or was determined from expert recommendations if an established MID was not available in published reports (Table 2).

**Fig. 2:**
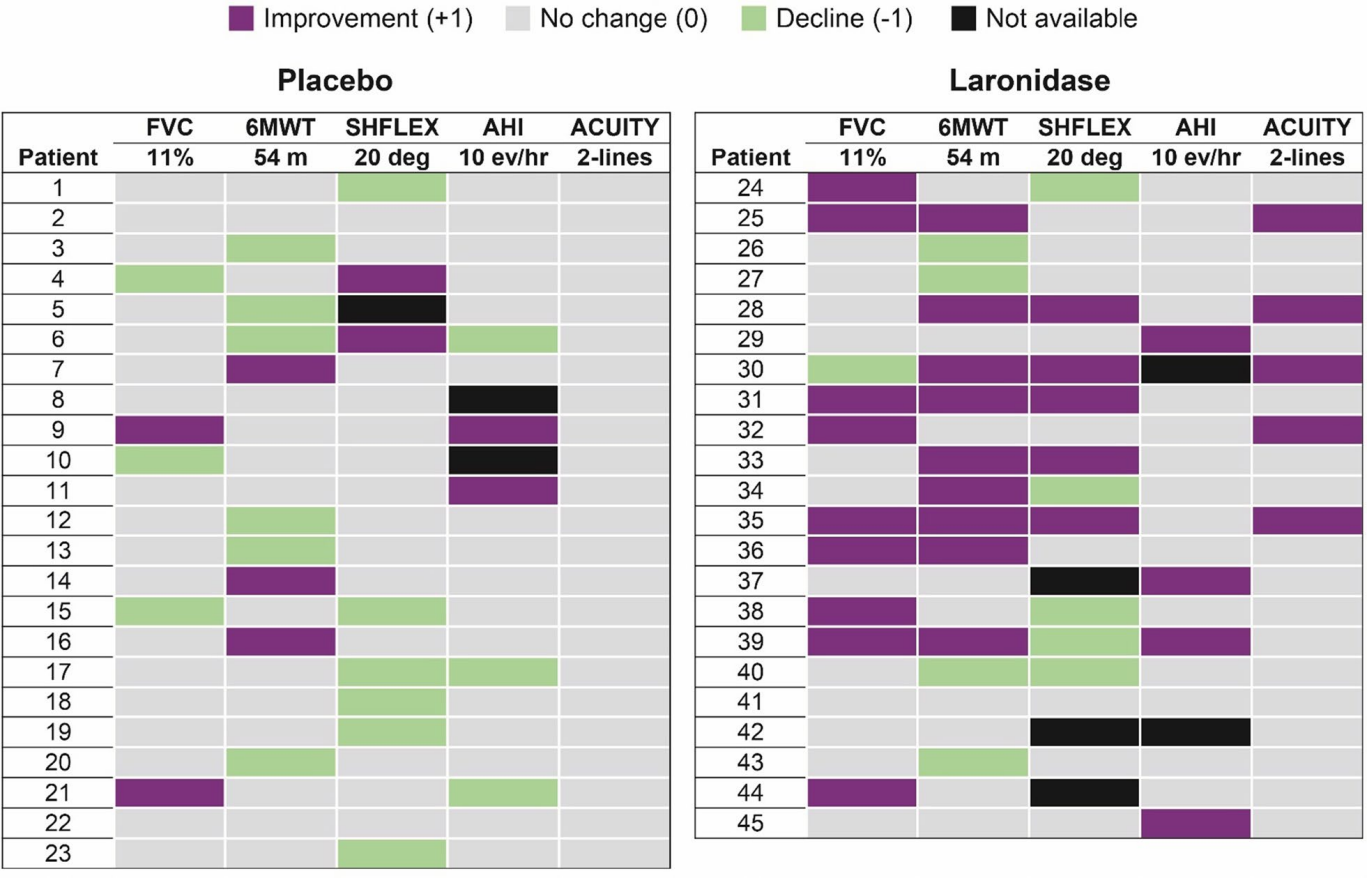
MDRI response using heat map approach in the Phase 3 Laronidase study

The five domains and MID were analyzed across 22 laronidase-treated patients and 23 placebo-treated patients and are presented in a heat map form in Fig. 2 where purple is + 1, green is − 1, tan is no change, and black is not available or not measurable. When observing the heat map presentation on a per patient basis, it is easy to see that green (− 1) outnumbers purple (+ 1) in the placebo group whereas purple (+ 1) greatly outnumbers green (− 1) in the laronidase group. The heat map shows eight laronidase-treated patients and only one placebo-treated patient with two or more + 1 domains. There are six laronidase-treated patients and no placebo-treated patients with three + 1 domains. Taken together, these results provide an assessment of the shift toward positive domain improvements in a broader and more insightful manner than any one endpoint.

**Fig. 3:**
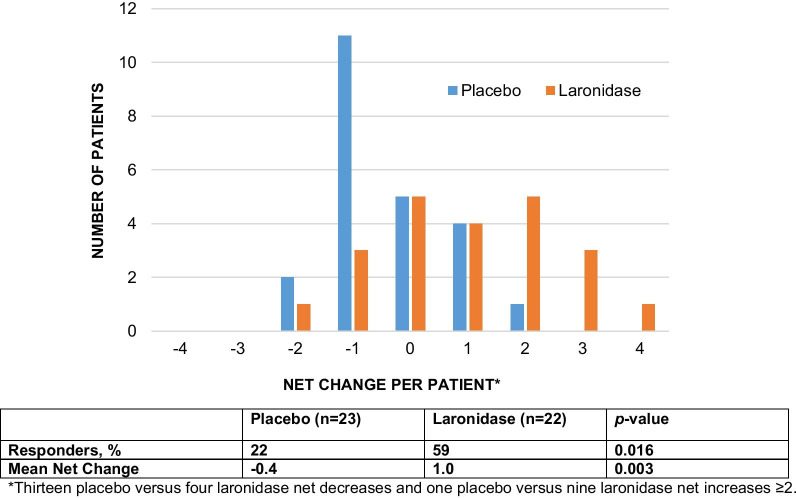
Net change in domains from a Phase 3 study of Laronidase versus Placebo

When the five domains were quantitatively assessed for MDRI response, there were more clinically significant improvements in the laronidase-treated patients than in the placebo-treated patients (Fig. 2). In patients treated with laronidase, 59% were net positive domain responders versus 22% of patients treated with placebo (*p* = 0.016). The mean net change in MDRI was + 1.0 for laronidase-treated patients versus − 0.4 for placebo-treated patients, a difference that was also statistically significant with *p* = 0.003 (Fig. 3).

The data also show the benefit of including three endpoints covering other disease manifestations in the analysis of efficacy, even when these abnormalities occur in 50% or fewer subjects. When looking vertically across the three additional endpoints, in the laronidase-treated group, there are 14 positive responses versus 4 decreases, and in the placebo group, there are 4 positive domain responses versus 9 decline responses, an eightfold difference in the ratio of positive to negative responses in favor of the laronidase-treated group. Due to the substantial baseline heterogeneity, these results were overlooked in the primary analysis of the study that was focused on the ITT analysis. These endpoints did not contribute to the original efficacy assessment, yet it is clear that these results are real and substantial contributions to the understanding of the laronidase treatment effect. The addition of these three additional endpoints broadens the assessment of efficacy and provides substantially more power than the two coprimary endpoints alone with a summary *p*-value for the MDRI that is tenfold smaller than for either co-primary endpoint alone. This should be expected since the mining of true additional power through the incorporation of additional orthogonal endpoints more completely captures the treatment effect and delivers the assessment with greater certainty than one or two endpoints.

#### MDRI in a study of Vestronidase Alfa in patients with MPS VII

The MDRI was provided as the key secondary clinical endpoint in a phase 3, randomized, placebo-controlled, blind-start, single crossover study of vestronidase alfa in 12 patients with MPS VII [17]. In this study, the MDRI consisted of the following clinical domains: 6MWT, FVC, shoulder flexion, visual acuity, and Bruininks–Oseretsky Test (BOT-2) fine motor proficiency, and BOT-2 gross motor proficiency. Since it was known that this disease was so rare that fewer than 50 patients had been identified alive in the developed world with this condition, enrolling a study of only 12 patients would require that the study sponsor essentially include all comers, regardless of disease stage at baseline. Since some older patients were already wheelchair bound due to degenerative hip disease at baseline, there was no way to measure walking distance or expect any improvement. Only six patients walked reliably in the study and the other six were not ambulatory consistently or at all. As such, shoulder flexion and the fine and gross motor tests were included to assure that some assessable domains in non-ambulatory patients were included in the study. In our experiences here and elsewhere, upper motor function is at least as important as walking given its importance in activities of daily living.

Similar to the analysis of the laronidase study described above, the MID for each domain was prospectively defined using the literature and experience from previous studies in patients with MPS (Table 3).

**Table 3:** MDRI domains and MID thresholds from the Phase 3 study of Vestronidase Alfa [17]

Domain	MID threshold
6MWT	23 m and ≥ 10% change from baseline [2, 3, 5, 6, 18–21]
FVC_%pred_	5% absolute change or ≥ 10% relative change from baseline in FVC_%pred_ [2, 6]
Shoulder flexion ROM	≥ 20 degree change of passive shoulder range of motion [2, 3, 5, 22]
Visual acuity	≥ 3 lines (corrected, both eyes) [23–25]
BOT-2 fine motor	Fine motor precision: ≥ change of 0.72
	Manual dexterity: ≥ change of 1.47 [26]
BOT-2 gross motor	Balance: ≥ change of 0.57
	Running speed and agility: ≥ change of 0.59 [26]

*6MWT* six minute walk test, *FVC*_*%pred*_ forced vital capacity percentage of predicted, *ROM* range of motion, *BOT-2* Bruininks–Oseretsky Test

The study was a novel, blind-start design in which each patient was randomized to one of four groups: one group started study drug at week 0, and all other groups began the study receiving placebo and then crossed over to active drug at weeks 8, 16, or 24. The final assessment for each patient was at week 24. The data for 48 weeks of total treatment was also analyzed. The primary analysis was a before versus after comparison with the MDRI included as the key secondary clinical endpoint. The primary endpoint for this small study was the urinary GAG substrate excretion in the urine, a biomarker that shows a strong relationship to tissue storage reduction [27]. In the extension study, the MDRI was the predefined key secondary endpoint.

**Fig. 4:**
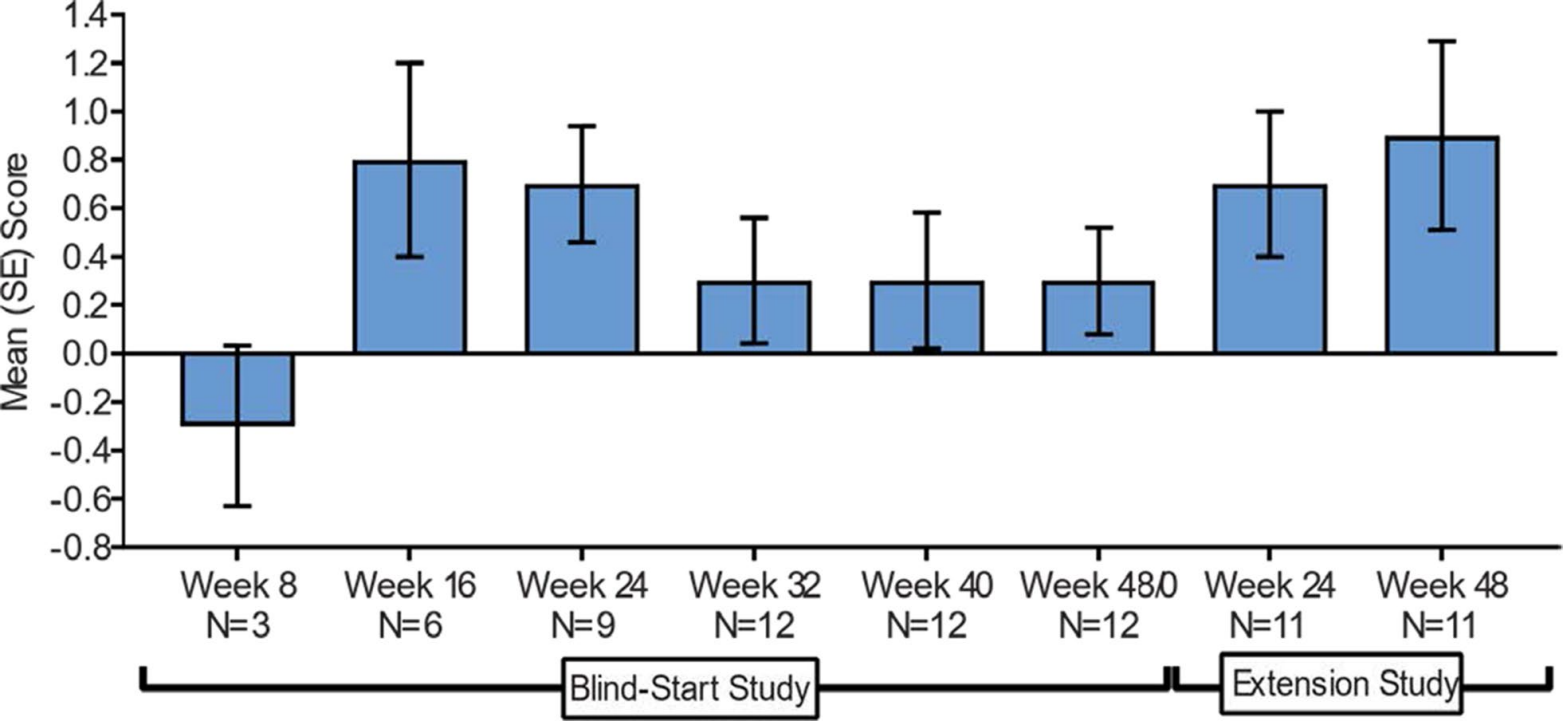
Mean (± SE) MDRI score during the Vestronidase blind-start and extension studies* [27]

**Fig. 5:**
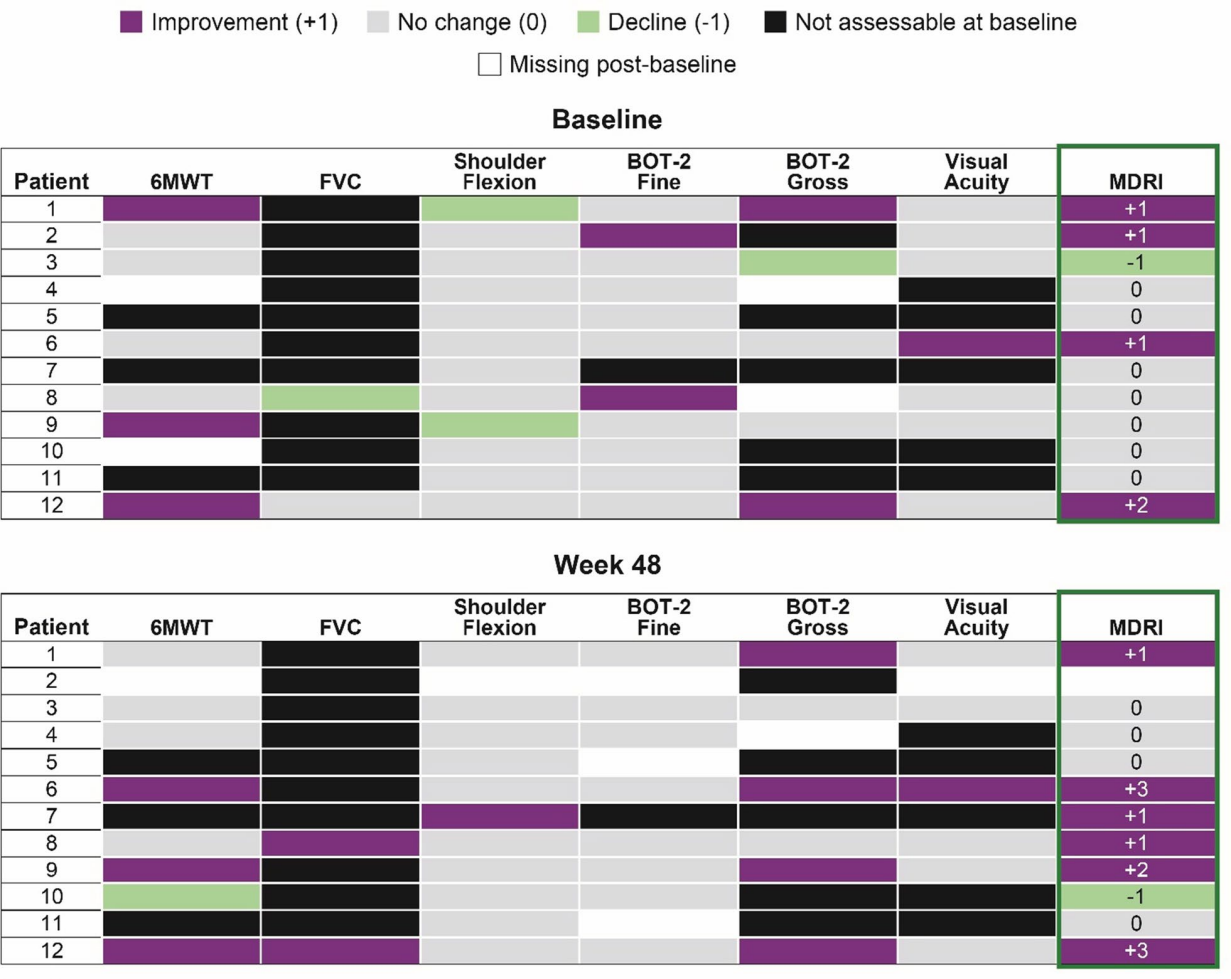
Patient-level MDRI at baseline and week 48 in the extension study* [27]

Figure 4 shows the mean domain score during the study conduct and Fig. 5 shows the heat map at baseline and at the Week 48 supplemental analysis. For the MDRI result, an overall mean (± SD) change of + 0.5 (± 0.8) at Treatment Week 24 (*p* = 0.052) was observed [17]. If the fatigue score (not shown) was included in the MDRI as originally proposed, another four net-positive responses would have been added, leading to a mean domain change of + 0.8 and p-value < 0.05. At the patient-level, 6 of 12 showed an improvement in MDRI total score of + 1 or more, and 5 of 12 had an MDRI score of 0, indicating potential stabilization of disease. Positive MDRI domains outnumbered negative domains 3:1, demonstrating an overall improvement of disease symptoms in this study population.

For a 12 patient, randomized, blind-start study, achieving this level of significance using a clinical responder analysis is impressive and was only possible in this heterogeneous population due to the ability of the MDRI to capture efficacy across different domains.

### The application of MDRI in neurologic disorders

Neurologic disorders, often associated with highly variable patterns of neurologic complications, are an ideal place for the use of the MDRI. A group of patients with the exact same biochemical disease can express a variety of findings and variable stages of disease. While neuronopathic MPS has a variety of central nervous system challenges (cognition, language, seizures, sleep disturbances), all could be considered clinically important. There are also a large number of complex single gene neurologic diseases in which MDRI could be used.

An example in neurologic diseases where the MDRI concept could be easily applied in which a diverse set of distinct manifestations exist that are all caused by a common CNS disease is Angelman syndrome, a rare, neurogenetic disorder caused by loss-of-function of the maternally inherited allele of the *UBE3A* gene. Individuals with Angelman syndrome can have developmental delay, almost universal loss of speech (with the majority of the patient population being completely non-verbal), gross motor dysfunction, ataxia/balance issues, fine motor limitations, severe sleep disturbances, and debilitating seizures [28, 29]. The expression of these manifestations can be widely variable between patients [28–30]. Some individuals with Angelman syndrome have difficulty with walking and balance, some do not respond to their name, and most do not speak any words. Some prefer to communicate with gestures or signs, others with sounds and communication devices, and some cannot communicate much at all [29–31]. Anxiety often significantly increases with age, and disturbed sleep can be a serious challenge in many individuals with Angelman syndrome, but not all [29, 32]. While these individuals have a normal lifespan, they require continuous care and are unable to live independently.

**Fig. 6:**
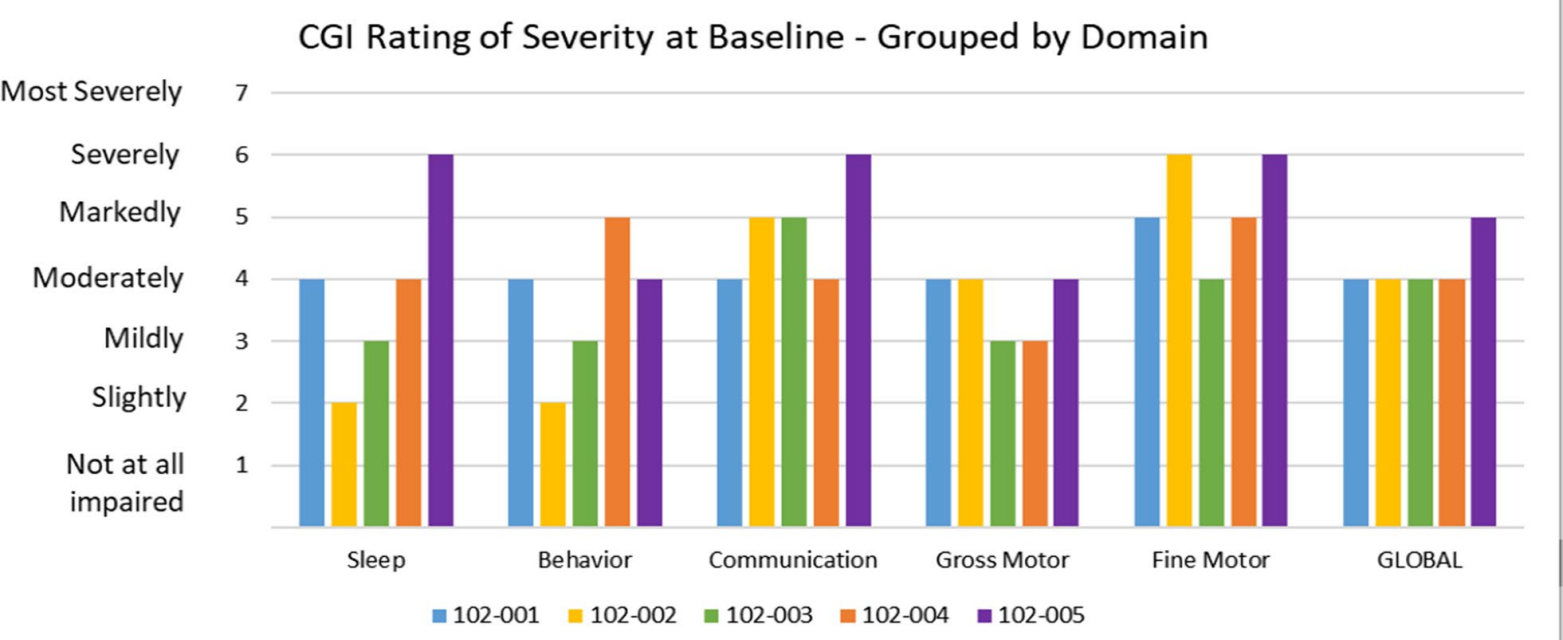
Clinical Global Impression (CGI) Scale rating of severity at baseline in Angelman syndrome

The impact of an antisense oligonucleotide, GTX-102, is currently being studied in our Angelman program across five important domains: communication, fine motor, gross motor, behavior, and sleep disturbance using individual endpoints for each. At baseline in the first five patients, there is a broad array of severity scores for each patient with communication impairments common in the more severe categories, but seizures, aberrant behaviors, and sleep disturbances can be quite profound and very variable, with some people no disturbance, others mild to moderate, and others severe. This baseline variation makes it hard to pick any particular primary endpoint without then restricting the population to that segment affected, and therefore we lose information and insight from the trial on the other manifestations. The unique palette of findings in each of these patients shows how hard it is to pick one endpoint to represent Angelman syndrome and certainly how hard it is to justify that one endpoint defines whether a treatment works or not (Fig. 6). While we have not yet utilized the MDRI for the Angelman program, this is an example of a situation where complex neurologic manifestations can be assessed using the MDRI for these five domains, and all types of patients can be included in the study and yet get a cogent and rigorous assessment of overall efficacy across five domains.

#### Comparison of MDRI with other instruments such as goal attainment scaling (GAS)

GAS is an instrument intended to evaluate the effect of an intervention by assessing change in daily life activities on an individual basis. However, GAS has not been validated adequately in the randomized controlled trial setting [33]. There are very clear differences in MDRI and GAS. In MDRI, the domains and the scoring (+ 1, 0, − 1) are fixed, whereas in GAS the items (goals) and scoring options (goal attainment levels) are different for each patient. The MDRI can be used for many patients and unbiased comparisons can be made between the treatment groups, whereas in GAS, patients make their own personalized instrument for one occasion in collaboration with their clinician. The other biggest difference between MDRI and GAS is that in the former, the score for each domain is ‘anchored’ to an MID and is interpretable on an individual domain level, whereas in GAS, the score is not ‘anchored’ and can only be interpreted as a comparison of group means in a randomized setting.

An additional challenge with GAS is the generalizability of the treatment efficacy results to other patients. For example, in MDRI, a mean difference of + 1 between two treatment group means that on average, patients are performing better in at least one domain in one treatment group compared with the other treatment group. This interpretation is easily generalizable with MDRI, and such a generalized interpretation is not possible in GAS.

### Key questions regarding MDRI and its interpretation

The interpretation of the overall results is straightforward. For example, if there is an overall change of + 1 in the analysis favoring experimental therapy (p < 0.01), this means that on average, the experimental therapy performed better than the control group in at least one domain out of X number of domains studied. This result is statistically reliable at a 0.01 level of significance. Similarly, an overall score of + 3 means that the experimental therapy performed better than placebo on 3 domains out of X domains studied.

#### Should responder index scores or continuous (or categorical) response variables be used for scoring each domain?

To combine domains, the use of the MID responder score assures that we are not adding up small changes to create meaningful changes. If we used continuous variables on each domain, we would lose the interpretability of the result and then the low prevalence of a problem within a domain would harm the result. The MID responder calculation also acts as a filter for nonconsequential changes that can dilute out real results, and this would be lost with a continuous variable analysis of each domain. In some ways, the MID responder scoring approach is better suited to the heterogeneity and whatever power is lost on each endpoint due to the responder analysis is made up for with the increased power of multiple domains.

#### Once domains are scored, what is the recommended method of analysis?

Our recommendation is to analyze net changes in MDRI scores using a non-parametric continuous variable analytical approach such as Wilcoxon Rank Sum test. It is well understood that, generally, a continuous variable analysis is more powerful than a binary analysis. It is also recommended to display the data using a responder analysis, where the responder is defined as a net change > 0, as shown in Fig. 3.

#### Does the limited + 1 scoring lose the value of higher levels of efficacy?

It is clear that strong responders would score only + 1 like a minimal clinically important change, and this will lose power that might come from the patients with larger changes. However, the addition of other endpoints will more than make up for this loss of power in most situations. An alternative scoring system which provides integer values above + 1, for example a + 2 score for a change that is 2× or greater than the MID is also possible. The proposal does not restrict the idea of using analyses more responsive to larger changes, but there is complexity in understanding how the endpoint is balanced with other endpoints in terms of equal balance if going to 2× MID is easier for one endpoint than another, for example. It is highly recommended to keep the same scoring scheme for all the domains under consideration. The scoring is certainly one area for further exploration in the use of the MDRI.

#### How can one equate pulmonary function with visual acuity accurately or any two endpoints?

The ability to verify that a + 1 in one domain is really the same as a + 1 in another domain, or more importantly that a − 1 in one domain is really fully balanced by a + 1 in another domain, is hard to verify and very subjective. Most would consider pulmonary function as more important than vision. But patients were often not aware of the impaired pulmonary function, but are more affected by their vision, and likely might score these differently. Establishing the relative value will never be perfect but neither is it perfect in picking one primary endpoint and ignoring or not powering others. Either way, choices are made as to the importance of endpoints and it is clear that no choice will be right for everyone. The careful construction of the MDRI with clearly meaningful endpoints of function or outcome can mitigate to some extent the situation where one superfluous endpoint is poised as equal to one with dire consequences. Since this is known ahead of time, it can be negotiated. Secondly, regardless of the results, positive or not, if one critical important domain has substantial declines in a study, and one that is good has increases, this fact will be obvious and known and a decision on the net benefit also evaluated independently, beyond the confines of the MDRI statistical analysis.

#### How are missing data imputed or not?

This is a really important topic that needs to be addressed as missing data are common in many clinical trials. There are three classes of missing data in clinical trials: (1) *missing completely random*, indicating that whether a data value is missing is completely unrelated to observed or unobserved data; (2) *missing at random*, indicating whether a missing value can be explained by the observed data; or (3) *missing not random*, meaning that the missingness is dependent on the unobserved values. When the missing data occur, it is important not to exclude patients with missing information. In the examples presented above, there were two types of missing data: “non assessable” which we will classify as missing not random. In these situations, patients really cannot perform the test, like the 6MWT. For the analysis purposes, we assigned a score of 0 (no change). It should be obvious that if there are many patients with nonrandom missing data, their contribution to the overall analysis is minimal, therefore a lower power of the study, but the analysis is valid.

The second type of missing data could be where a patient can perform the test at baseline, but there are some missing values for various reasons (*eg*, a missing visit). These types of data are generally classified as missing at random. The proper way of handling missing at random data are multiple imputation techniques [34]. Once the missing data are imputed, the MDRI methodology can easily be applied to the dataset.

#### Why do we allow patients in the study that cannot do all the tests?

The common problem in enrolling studies of rare diseases is finding patients of all ages that can do all the tests. While many patients, for example those less than 6 or 7 years old, have serious disease, conducting the pulmonary function testing for FVC correctly and accurately is very difficult. These same patients might have terrible joint restriction as observed in the laronidase program. If we require all patients to do all the tests, we immediately set age, disease level and other requirements which narrow the population, limit enrollment, and more significantly, limit insight into a disease. The mindset focused on no missing data and obsession with perfection leads to imperfect assessments of disease in limited, highly crafted population subsets. It is time to break with our historic view and see trials and endpoints as adapting to the clinical need and enhancing the power and breadth of our clinical insight. That is closer to perfection than what we are doing today.

#### How do we label a product that has only, for example, 3 domains out of 6 actually contributing to the result?

The MDRI analysis provides the hypothesis test and statistical rigor for determining that substantial efficacy exists. Once that determination is made, the question can be asked as to what is the basis for this efficacy. By analyzing the MDRI results, it may turn out that three domains dominate the efficacy and for reasons unpredicted, patients did not have other abnormalities at baseline, or the disease was not reversible. The sensitivity analysis, by removing individual domains from the MDRI and assessing the impact of each on statistical significance and treatment effect estimates would allow the analyst to then answer the question of which domains drove the MDRI positive result. The labeling would then be based on those domains significantly contributing. The criteria for this analysis and threshold for significance can be predefined in the statistical analysis plan. In some cases, it is obvious, as in the laronidase case where FVC, 6MWT, Shoulder ROM, and AHI all occurred in 50% or more of subjects and had numerous responders. Visual acuity was much less common at 18%, but the impact in five patients was very large. The case for a finding like visual acuity would need to be predefined and agreed to with regulators, with two questions of importance: (1) are there enough responders in the assessed population to be confident of its interpretation? And (2) Is this effect in absolute terms, not just in responder MID units, substantial enough to be labeled? For visual acuity the number was small, with 5 versus 3 compared in a total 45 patient study, but the effect size of more than 4 lines of improvement in visual acuity for every single patient is so distinct, with no change in 3 placebo-treated patients, to consider this as part of the labeling.

## Conclusions

The MDRI is a method of analysis that can effectively capture the totality of clinical trial data in a rigorous fashion and summarize these data in a reasonable and powerful manner. Complex, variable multi-system disorders with heterogeneous disease manifestations, such as MPS diseases, may benefit from such an efficacy assessment approach where the prevalence of specific symptoms or disease manifestations may be less than 50% and yet are serious and important. A greater acceptance of a broad array of patients eligible for clinical studies provides a far better evaluation of safety and greater potential to understand the impact of treatment across a range of disease severity compared with the crafted segments of patient populations compelled by single primary endpoint designs.

With the MDRI, the built-in clinical meaningfulness thresholds establish a level of efficacy within a patient, and avoid the inclusion of small, insignificant changes in the overall efficacy assessment, and the problem of multiplicity of analyses for multiple endpoints that impairs most rare disease studies is eliminated. The need to develop and validate a novel composite endpoint is eliminated, and the limitation of composites in their use in multisystem diseases is avoided. Limitations of the MDRI may include that the selected endpoints do not reflect the most impactful clinical endpoints that patients would select when using an endpoint paradigm such as GAS; however, choosing the appropriate endpoints and establishing the degree of beneficial improvements are both challenges that may prevent trials with GAS endpoints from establishing efficacy for agents that otherwise may have been beneficial.

Further study of best scoring analysis methods for the MDRI (+ 1, 0, − 1, or integers over the MID) could help in specific situations. We believe that the adoption of the MDRI approach as the primary endpoint for the determination of overall efficacy will be a game-changer for rare disease clinical development, allowing better, broader and more meaningful studies of rare diseases to be conducted faster and better, without the fear that a random variation or error in a choice will lead to disaster for the study and the patients that might have benefited from the drug.

## Data Availability

The datasets used and analyzed in the current study are available from the corresponding author on reasonable request.

## Abbreviations

AHI: Apnea–Hypopnea index
ANCOVA: Analysis of covariance
BOT-2: Bruininks–Oseretsky test
CHAQ: Child Health Assessment Questionnaire
FVC: Forced vital capacity
GAGs: Glycosaminoglycans
HAQ: Health Assessment Questionnaire
ITT: Intent-to-treat
MDRI: Multi-domain responder index
MID: Minimally important difference
MPS I: Mucopolysaccharidosis type I
NSAA: North Star Ambulatory Assessment
ROM: Range of motion
6MWT: Six minute walk test

## References

[1] Greene JA, Podolsky SH (2012). Reform, regulation, and pharmaceuticals–the Kefauver–Harris Amendments at 50. N Engl J Med.

[2] Wraith JE (2004). Enzyme replacement therapy for mucopolysaccharidosis I: a randomized, double-blinded, placebo-controlled, multinational study of recombinant human alpha-l-iduronidase (laronidase). J Pediatr.

[3] Harmatz P (2006). Enzyme replacement therapy for mucopolysaccharidosis VI: a phase 3, randomized, double-blind, placebo-controlled, multinational study of recombinant human *N*-acetylgalactosamine 4-sulfatase (recombinant human arylsulfatase B or rhASB) and follow-on, open-label extension study. J Pediatr.

[4] Kakkis ED (1996). Long-term and high-dose trials of enzyme replacement therapy in the canine model of mucopolysaccharidosis I. Biochem Mol Med.

[5] Clarke LA (2009). Long-term efficacy and safety of laronidase in the treatment of mucopolysaccharidosis I. Pediatrics.

[6] Muenzer J (2006). A phase II/III clinical study of enzyme replacement therapy with idursulfase in mucopolysaccharidosis II (Hunter syndrome). Genet Med.

[7] Chi GY (2005). Some issues with composite endpoints in clinical trials. Fundam Clin Pharmacol.

[8] Sankoh AJ, Li H, D'Agostino RB (2014). Use of composite endpoints in clinical trials. Stat Med.

[9] Garcia-Garcia HM (2018). Standardized end point definitions for coronary intervention trials: the academic research consortium-2 consensus document. Circulation.

[10] Mazzone ES (2009). Reliability of the North Star Ambulatory Assessment in a multicentric setting. Neuromuscul Disord.

[11] Mayhew J (2018). Development and preliminary evidence of the psychometric properties of the GNE myopathy functional activity scale. J Comp Eff Res.

[12] Vockley J (2019). Results from a 78-week, single-arm, open-label phase 2 study to evaluate UX007 in pediatric and adult patients with severe long-chain fatty acid oxidation disorders (LC-FAOD). J Inherit Metab Dis.

[13] Balwani M (2020). Phase 3 trial of RNAi therapeutic Givosiran for acute intermittent porphyria. N Engl J Med.

[14] O'Brien PC (1984). Procedures for comparing samples with multiple endpoints. Biometrics.

[15] Pocock SJ, Geller NL, Tsiatis AA (1987). The analysis of multiple endpoints in clinical trials. Biometrics.

[16] Tandon PK (1990). Applications of global statistics in analysing quality of life data. Stat Med.

[17] Harmatz P (2018). A novel Blind Start study design to investigate vestronidase alfa for mucopolysaccharidosis VII, an ultra-rare genetic disease. Mol Genet Metab.

[18] Redelmeier DA (1997). Interpreting small differences in functional status: the Six Minute Walk test in chronic lung disease patients. Am J Respir Crit Care Med.

[19] Puhan MA (2008). Interpretation of treatment changes in 6-minute walk distance in patients with COPD. Eur Respir J.

[20] du Bois RM (2011). Six-minute-walk test in idiopathic pulmonary fibrosis: test validation and minimal clinically important difference. Am J Respir Crit Care Med.

[21] Mathai SC (2012). The minimal important difference in the 6-minute walk test for patients with pulmonary arterial hypertension. Am J Respir Crit Care Med.

[22] Okuyama T (2010). Japan Elaprase Treatment (JET) study: idursulfase enzyme replacement therapy in adult patients with attenuated Hunter syndrome (Mucopolysaccharidosis II, MPS II). Mol Genet Metab.

[23] *Photodynamic therapy of subfoveal choroidal neovascularization in age-related macular degeneration with verteporfin: one-year results of 2 randomized clinical trials--TAP report. Treatment of age-related macular degeneration with photodynamic therapy (TAP) Study Group.* Arch Ophthalmol, 1999. 117(10): p. 1329–45. 10532441

[24] Ferris FL (1982). New visual acuity charts for clinical research. Am J Ophthalmol.

[25] Reeves BC, Wood JM, Hill AR (1993). Reliability of high- and low-contrast letter charts. Ophthal Physiol Opt.

[26] Wuang YP, Su CY (2009). Reliability and responsiveness of the Bruininks–Oseretsky test of motor proficiency-second edition in children with intellectual disability. Res Dev Disabil.

[27] Wang RY (2020). The long-term safety and efficacy of vestronidase alfa, rhGUS enzyme replacement therapy, in subjects with mucopolysaccharidosis VII. Mol Genet Metab.

[28] Dagli, A.I., J. Mueller, and C.A. Williams, *Angelman Syndrome*, in *GeneReviews((R))*, M.P. Adam, et al., Editors. 1993: Seattle (WA).

[29] Wheeler AC, Sacco P, Cabo R (2017). Unmet clinical needs and burden in Angelman syndrome: a review of the literature. Orphanet J Rare Dis.

[30] Keute M (2020). Angelman syndrome genotypes manifest varying degrees of clinical severity and developmental impairment. Mol Psychiatry.

[31] Gentile JK (2010). A neurodevelopmental survey of Angelman syndrome with genotype-phenotype correlations. J Dev Behav Pediatr.

[32] Willgoss T (2020). Measuring what matters to individuals with Angelman syndrome and their families: Development of a patient-centered disease concept model. Child Psychiatry Hum Dev.

[33] Gaasterland CMW (2019). Goal attainment scaling as an outcome measure in rare disease trials: a conceptual proposal for validation. BMC Med Res Methodol.

[34] Little RJA, Rubin DB (2002). Statistical Analysis with Missing Data.

